# DICAR/DICAR-JP exerts therapeutic effects in brain stroke via the miR-361-5p/PRMT1 pathway

**DOI:** 10.3389/fphar.2025.1721188

**Published:** 2025-11-27

**Authors:** Zhijun Yu, Aihua Jiang, Kai Yang, Lin Zhan, Zifei Xiang, Li Chen, Zheng Kuai, Qiong Yuan

**Affiliations:** 1 Pharmacy, Medical College, Wuhan University of Science and Technology, Wuhan, China; 2 Hubei Province Key Laboratory of Occupational Hazard Identification and Control, Medical College, Wuhan University of Science and Technology, Wuhan, China; 3 Key Laboratory of Medical Electrophysiology, Ministry of Education & Medical Electrophysiological Key Laboratory of Sichuan Province Collaborative Innovation Center for Prevention of Cardiovascular Diseases, Institute of Cardiovascular Research, Southwest Medical University, Luzhou, China; 4 Department of Geriatrics, Zhongshan Hospital, Fudan University, Shanghai, China

**Keywords:** stroke, diabetes-induced circulation-associated circular RNA, RNA functional domain, angiogenesis, miR-361-5p, PRMT1

## Abstract

**Background:**

Angiogenesis is an important mechanism in stroke therapy. Circular RNA-DICAR is known to protect against diabetes-induced cardiomyocyte pyroptosis. In this study, we examined the effect of DICAR on angiogenesis as well as the therapeutic effect of DICAR-JP, which is its functional domain, against stroke. The mechanism involves the miR-361-5p/PRMT1 signaling pathway. Moreover, the middle cerebral artery occlusion/reperfusion (MCAO) method was used to establish a mouse stroke model in the DICAR-Tg mouse model.

**Methods:**

AAV9-DICAR-JP was constructed and injected into the cerebral ventricles. Furthermore, tube formation assays were used to evaluate the *in vitro* activity of DICAR-JP. The blood flow was tested by Laser Speckle Flowgraphy. The expressions of PRMT1 protein expression was evaluated by Western blotting (WB). The binding ability was evaluated by luciferase reporter.

**Results:**

Results indicated that DICAR-Tg improved neuronal function. AAV9-DICAR-JP increased blood flow in the brain following stroke. DICAR-siRNA upregulated miR-361-5p expression, and overexpression of miR-361-5p significantly reduced PRMT1 expression.

**Conclusion:**

DICAR/DICAR-JP has potential therapeutic benefits in stroke, which is mediated by the miR-361-5p/PRMT1 signaling pathway.

## Introduction

The blood–brain barrier (BBB)—a key component of the neurovascular system—regulates cerebral microcirculation during ischemic stroke (Lyu et al., 2025; Gao et al., 2025). This specialized interface modulates arterial microcirculation to enhance cerebral oxygen extraction under hypoxic conditions (Zhang et al., 2019). Angiogenesis or the formation of new blood vessels from existing ones, is crucial for physiological balance as well as disease progression (Yuan et al., 2025; Kayan and Savas, 2025). Intermittent hypoxia conditioning can protect against hypoxic-ischemic encephalopathy by promoting angiogenesis (Ye et al., 2022). Cerebrovascular endothelial cells (ECs) maintain vascular stability by producing vasoactive mediators, such as nitric oxide (NO), which attenuate vascular tone and cerebral blood flow. Thus, elucidating the changes in EC signaling pathways may provide insight for the development of new treatments for cerebrovascular disorders.

Circular RNAs (circRNAs) act as key regulators of angiogenesis in cerebrovascular pathophysiology (Bai et al., 2018; Qi et al., 2025; Jia et al., 2025). Thus far, two circRNAs that modulate angiogenesis have been functionally characterized. Notably, circRNA_0003307 promotes cerebral ischemia–reperfusion injury by enhancing brain microvascular endothelial cell angiogenic activity and metastasis, possibly through the miRNA-191-5p/CDK6 signaling axis (Zhang et al., 2019). Conversely, increased circPDS5B expression worsens ischemic stroke through hnRNPL-mediated stabilization of the Runx1/ZNF24 transcriptional complex, resulting in the suppression of VEGFA-mediated angiogenic signaling. We previously demonstrated that DICAR acts as a cardioprotective circRNA that mitigates cardiomyocyte pyroptosis during diabetic cardiomyopathy (Yuan et al., 2023). Studies have shown that brain microvascular endothelial cells undergo pyroptosis after ischemic stroke (Wang et al., 2021). Notably, endothelial cell pyroptosis exacerbates BBB disruption, allowing various pro-inflammatory factors to enter the brain parenchyma, further aggravating brain injury (Long et al., 2023; Cheng et al., 2026). Based on the effect of DICAR on the anti-pyroptosis, and the relationship of pyroptosis and angiogenesis, maybe DICAR also belong the function on the aniogenesis of endothelial cells. Therefore, in the present study, we aim to elucidate the potential regulatory relationship between DICAR expression and cerebrovascular ischemic pathogenesis.

A well-known function of circRNAs is their role as molecular sponges that sequester microRNAs (miRNAs), thus reducing miRNA-mediated post-transcriptional regulation of target genes (2020, Liu et al., 2019). For example, circ_0001142 binds to miR-361-3p, disrupting autophagic flux and reprogramming macrophage phenotypic switching. This regulation depends on PIK3CB, a downstream effector gene targeted by miR-361-3p for polarization control (Memczak et al., 2013). Moreover, circ_0006089 has an oncogenic role in gastric cancer (GC). It is significantly upregulated in GC tissues and cell lines, where it acts as a molecular sponge for miR-361-3p. Notably, silencing circ_0006089 inhibits tumor growth, metastasis, glycolytic flux, and angiogenesis while promoting apoptosis. These effects are reversible by miR-361-3p inhibition (Zhou et al., 2022). The tumor-suppressive miR-361-3p directly targets TGFB1, a key regulator of cancer cell plasticity. Moreover, rescue experiments revealed that overexpressing TGFB1 counteracts the miR-361-3p-mediated suppression of oncogenic activity in GC cells. This establishes the miR-361-3p/TGFB1 axis as an important factor in GC pathobiology (Zhou et al., 2022).

In the present study, we examined DICAR/DICAR-JP function in stroke and its potential underlying mechanism.

## Methods and materials

### Animals and the middle cerebral artery occlusion/reperfusion (MCAO) model

The methods used were similar to those described previously (Zhang et al., 2019). Male and female C57BL/KsJ wild-type (WT) (age: 12 weeks) and C57BL/KsJ db/db mice with 23–28 mM blood glucose levels were procured from the GenePharmatech Company. DICAR-Tg mice were established in our laboratory (Yuan et al., 2023). All animal studies were conducted with age- and gender-matched controls, and the mice were maintained in a temperature-controlled (22 °C–25 °C) environment under a 12-h light/dark cycle with free access to food and water at the Animal Center of Wuhan University of Science and Technology. Adult male C57/BL6 and DICAR-Tg mice [Certificate No: SCXK (Q) 2015–0018; 18–25 g, 1.5–2.0 months old] were bred at the Wuhan University of Science and Technology’s Experimental Animal Center. They were maintained under the same conditions as described above. Focal cerebral ischemia–reperfusion (I/R) injury was induced by transient right MCAO for 1 h, followed by reperfusion for 0, 6, 12, or 24 h. The mice were randomly assigned to sham (n = 16) or MCAO model groups (n = 60). The animal research adheres to the double-blind principle in all animal experiments. Briefly, mice were deeply anesthetized using a 1%−2% isoflurane oxygen/nitrous oxide mixture in a ratio of 30% and 69% administered through a mask applied to the face; body temperature was maintained at 37 °C + 0.3 °C with a small animal heating platform. The left common carotid as well as the external and internal carotid arteries were exposed and a silicone-coated 6-0 suture was routed from the stump of the external to the internal carotid artery until the lumen of the middle cerebral artery was reached. The distances from the bifurcation of the internal and external carotid artery to the middle cerebral artery was 10 + 0.5 mm. Laser Doppler Flowmetry (Moor Instruments, UK) was used to establish that occlusion had been successfully achieved. The same procedure was used for sham-operated animals with the exception that the suture was routed along the internal carotid artery before being immediately withdrawn. Subsequently, neurological deficits were evaluated using the Longa method, with scores of 2 or 3 considered successful. Next, brain tissue was harvested and either snap-frozen for molecular analysis or fixed for morphological studies. All protocols and procedures performed in this study were approved by the Institutional Animal Care and Use Committee of the Wuhan University of Science and Technology (Approval Nos. 2017001), adhered to National Institute of Health Guide for the Care and Use of Laboratory Animals (2011), and reported according to ARRIVE guidelines 2.0.

#### Behavioral assessment

Behavioral assessments were conducted 24 h after reperfusion using Longa’s five-point neurological scoring system, as follows: 0-no detectable neurological deficits; 1-mild neurological impairment, evidenced by incomplete extension of the left forepaw; 2-moderate impairment, characterized by decreased resistance to lateral push and leftward circling; 3-severe impairment, evidenced by falling to the left; and 4-profound impairment, no spontaneous locomotion and markedly reduced consciousness level.

### TTC staining

Mice were anesthetized 24 h following reperfusion, and their brains were harvested and sectioned into 2-mm coronal slices, incubated with 2% 2,3,5-triphenyltetrazolium chloride (TTC) (Sigma, St. Louis, MO, United States) for 30 min at 37 °C in the dark, and fixed in 4% paraformaldehyde. The sections were removed, and the ischemic area (pale) was analyzed using ImageJ software (NIH Image, Version 1.61). The percentage of the brain-infarct volume was calculated as follows: infarct volume/total volume × 100% (Zhang et al., 2019).

## Cell culture and oxygen-glucose deprivation/reperfusion (OGD/R) model

hCMEC/D3 cells were cultured in Endothelial cell Medium (ECM, Gibco, Grand Island, NY, United States) supplemented with 10% fetal bovine serum (Gibco, Grand Island, NY, UNITED STATES) at 37 °C in a humidified incubator with 5% CO2. The cells were seeded in 6-well plates at a density of 3 × 105 cells per well. After 24 h, the medium was replaced with serum-free and glucose-free medium (Gibco). Subsequently, the plates were placed in a hypoxic chamber with a gas mixture of 95% N2 and 5% CO2 at 37 °C. After 3 h of oxygen-glucose deprivation (OGD), the medium was replaced with normal medium. Control cells did not receive OGD treatment. The cells were collected at 0, 6, 12, and 24 h of reperfusion for morphological assessment, qPCR, and Western blot analysis. The cells were seeded at 5 × 104 cells per well in 24-well plates 24 h before transfection. They were then transfected with DICAR-JP and a negative control (Ribobio, Guangzhou, Guangdong, China) using Lipofectamine RNA Mix (Invitrogen, Carlsbad, CA, United States) (Zhang et al., 2019).

### Cell transfection

For 24 h before OGD, cells were placed (3 × 105 cells) in 6-well plates and transfected with a negative control, miR-361-3p mimic (5′-p-UUUAGUCUUAGUGUGGACCCCCU-dTdT-3′), PRMT-OE (Miaolin, China) or DICAR-JP (sequences see Supplementary Table S1) by jetPRIME (Polyplus, Parc Eurasanté Ouest, France). After we detected the inhibitory effect of miR-361-3p mimic, was chosen for the next experiments and construction of PRMT-OE. After 48 h, cells were harvested for western blotting or were treated with OGD/R.

### Western blot analysis

Protein expression was determined through Western blot analysis. Brain tissue was homogenized in RIPA lysis buffer (Beyontime, Jiangsu, China) supplemented with 0.1 mM phenylmethylsulfonyl fluoride (PMSF) (Sigma, Missouri, United States) for immunoblotting analysis. The cells were harvested and homogenized in lysis buffer (50 mM Tris-HCl, pH 7.4, 150 mM NaCl, 1.5 mM MgCl2, 10% glycerol, 1% Triton X-100, 5 mM EGTA, 20 μM leupeptin, 1 mM AEBSF, 1 mM NaVO3, 10 mM NaF, and 1× protein inhibitor cocktail). The proteins were separated via sodium dodecyl sulfate-12% polyacrylamide gel electrophoresis (SDS-PAGE) on 12% gels and transferred to polyvinylidene fluoride (PVDF) membranes at 300 mA for 1.5 h. The membranes were blocked in TBS/T buffer (20 mM Tris-HCl, pH 7.6, 150 mM NaCl, 0.1% Tween-20) containing 5% non-fat milk at 37 °C for 2 h. The primary antibodies included rabbit anti-VEGF (1:1000, ab115319, Abcam, MA, United States), rabbit anti-VEGFR2 (1:1000, 9662S, Cell Signaling Technology, MA, United States), and mouse anti-β-actin (1:1000, sc-47778, Santa Cruz Biotechnology, CA, United States). All of the antibodies were diluted in Tris-Buffered Saline with Tween 20 (TBST) buffer (50 mM Tris-HCl, 150 mM NaCl, 0.1% Tween-20, pH 7.4) and incubated with the PVDF membranes at 4 °C overnight. Subsequently, corresponding horseradish peroxidase (HRP)-conjugated secondary antibodies (1:5000, A21010, Abbkine, CA, United States) were incubated with the PVDF membrane for 90 min at room temperature. Signal detection was done using an enhanced chemiluminescent (ECL) reagent (Amersham Biosciences, Piscataway, NJ, United States). The signals were detected with a Bio-Rad ChemiDoc MP system (Bio-Rad, Richmond, CA, United States).

### Real-time RT-PCR analysis

The expression of DICAR and GAPDH mRNA in brain tissue and hCMEC cells was analyzed using the CFX96 Real-Time PCR Detection System (Bio-Rad). Total RNA (1 μg) from each group was reverse-transcribed using the First Strand cDNA Synthesis Kit (Thermo Fisher), and PCR amplification was performed with SYBR Green Real-time PCR Master Mix (Takara) and 0.4 μM of each primer. The PCR protocol was as follows: initial step at 95 °C for 3 min, followed by 40 cycles of 95 °C for 30 s, 60 °C for 60 s, and 72 °C for 10 s. The reactions were run in triplicate, and the DICAR/GAPDH (primer sequecnes see Supplementary Table S2) ratio was calculated, with the control group set to 1.

### Dual-luciferase reporter gene assay

The dual-luciferase reporter gene assay was performed according to established protocols (Hu et al., 2020). Specifically, two putative miR-361-3p binding sites within the DICAR 3′UTR (Position 1: 112–118; Position 2: 218–224) were identified through bioinformatic analysis and subjected to site-directed mutagenesis. WT and mutant (MT) DICAR sequences were cloned into GV272 luciferase reporter vectors (GeneChem, Shanghai, China), and the miR-361-3p sequence was subcloned into the GV251 expression vector (GeneChem). For functional validation, 293T cells plated in 24-well plates were co-transfected with either 0.6 μg GV251-miR-361-3p plasmid or a negative control plasmid with 0.2-μg WT or MT reporter constructs. Luminescence signals were measured 48 h post-transfection using the Dual-Luciferase Reporter Assay System (Promega, Madison, WI, United States) following the manufacturer’s instructions. All experiments were conducted in triplicate with independent biological replicates.

### Laser Speckle Flowgraphy blood flow measurements

Measurements were conducted using the LSFG-NAVI system (Softcare Co. Ltd., RWD, China). This device visualizes the real-time velocity distribution of scattered particles in the ocular fundus, or the fundus blood flow distribution. We assessed the MBR of the large vessels at the optic nerve head. A rubber band (analysis area) was set as an ellipse along the inner edge of the optic nerve papilla, and the vascular area was selected using software. The mean background tissue area was subtracted from the mean vascular area to obtain the MBR of the papillary large vessels. The mean MBR of the large vessels of the optic papilla was used in this study because it reflects the circulation of the entire retina.

### Statistical analysis

Quantitative data were derived from a minimum of three independent experiments. The data are presented as the mean ± SD. Statistical analyses were performed by a one-way ANOVA with Tukey’s *post hoc* test for multiple comparisons using GraphPad Prism software (version 5.0). A p-value <0.05 was considered statistically significant.

## Results

### DICAR overexpression inhibits the impairment of cerebral neurons caused by brain ischemia/reperfusion over short and long periods

**FIGURE 1:**
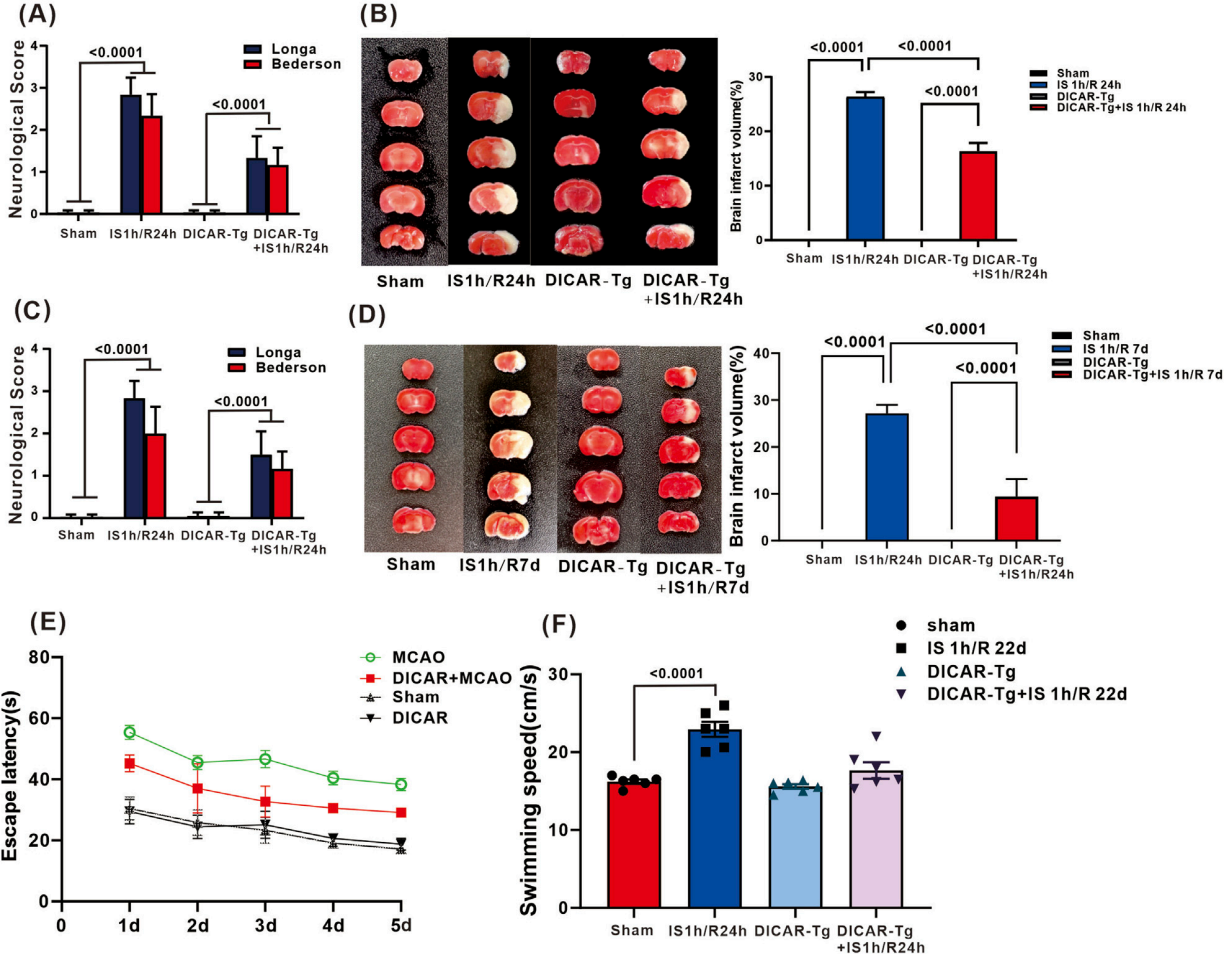
DICAR-Tg inhibited the acute injury and long-term of cerebral neurons induced by brain ischemia/reperfusion. **(A)** Average neurological scores of the Longa and Bedeson of brain IS 1h/R24 h. **(B)** Representative images and Statistical chart for TTC staining of brain IS 1 h/R24 h. **(C)** Average neurological scores of the Longa and Bedeson of brain IS 1 h/R7D; **(D)** Representative images and Statistical chart for TTC staining of brain IS 1 h/R7D. **(E)** the escape latency. **(F)** swimming speed and the target quadrant time tests statistical evaluation of the Morris water maze in brain IS1 h/R22d. Values represent the mean ± SD (n = 6 for each group).

DICAR expression was detected in the mouse brain I/R model. As illustrated in Supplementary Figure S1, DICAR was significantly increased after 1 h of ischemia (p < 0.0001); however, it was decreased at 1 h ischemia/6 h reperfusion (p < 0.0001), and then its expression level recovered (Supplementary Figure S1). To examine the role of DICAR, we established a DICAR knock-in mouse model and induced brain ischemia–reperfusion (I/R) for 1, 7, and 22 days. To determine the effect of DICAR on brain injury following I/R, 2,3,5-triphenyltetrazolium chloride (TTC) staining was used to measure the infarct size at 1 and 7 days, and behavioral assessments were done to evaluate cortical neuron function at 22 days. Neuronal function was considerably impaired following 1 h ischemia/24 h reperfusion (score: 2.8 ± 0.2, p < 0.0001; Figure 1A); however, DICAR overexpression significantly improved neurological function (score: 1.8 ± 0.3, p < 0.0001; Figure 1A). Compared with the 1 h ischemia/24 h reperfusion + WT group, DICAR overexpression significantly reduced the cortical infarct size (26.3% ± 0.9% vs. 16.3% ± 0.9%) by approximately 22% (p < 0.0001; Figures 1B,C). We also observed a protective effect at 7 days. The results indicate that DICAR-Tg protects neuron function after 1 h ischemia/7 days reperfusion (Long score: 1.5/7 daysBederson 1.17 on 1.17/7 days reperfusion (Lct at 7 days. The results.8 8cantly replicates. dison, WI, United States) Bederson 2.00 ± 0.26; p < 0.001; Figure 1C). DICAR-Tg + 1 h ischemia/7 days reperfusion also exhibited a smaller infarction area (9.42% ± 1.83%) compared with WT + 1 h ischemia/7 days reperfusion (27.14% ± 1.52%, p < 0.001; Figures 1D,E).

To evaluate the long-term effects of DICAR in cerebral I/R, the Morris water maze test was used to evaluate memory impairment. All groups exhibited decreased escape latencies with repeated trials. Notably, the DICAR-Tg + IS 1 h/R 22 d group had significantly longer escape latencies compared with the sham group (Figure 1E), indicating impaired spatial learning. In addition, the swimming ability of the WT IS 1 h/R 22 d group was impaired, whereas DICAR-Tg improved movement ability post-stroke (p < 0.0001, Figure 1F). We also established a DICAR^+/−^ mouse and assessed the recognition capacity of the mouse. As illustrated in Supplementary Figure S1, the recognition capacity of DICAR^+/−^ mouse was not impaired. These results showed that DICAR downregulation by itself did not impair brain function.

### DICAR-Tg increases cerebrovascular density

**FIGURE 2:**
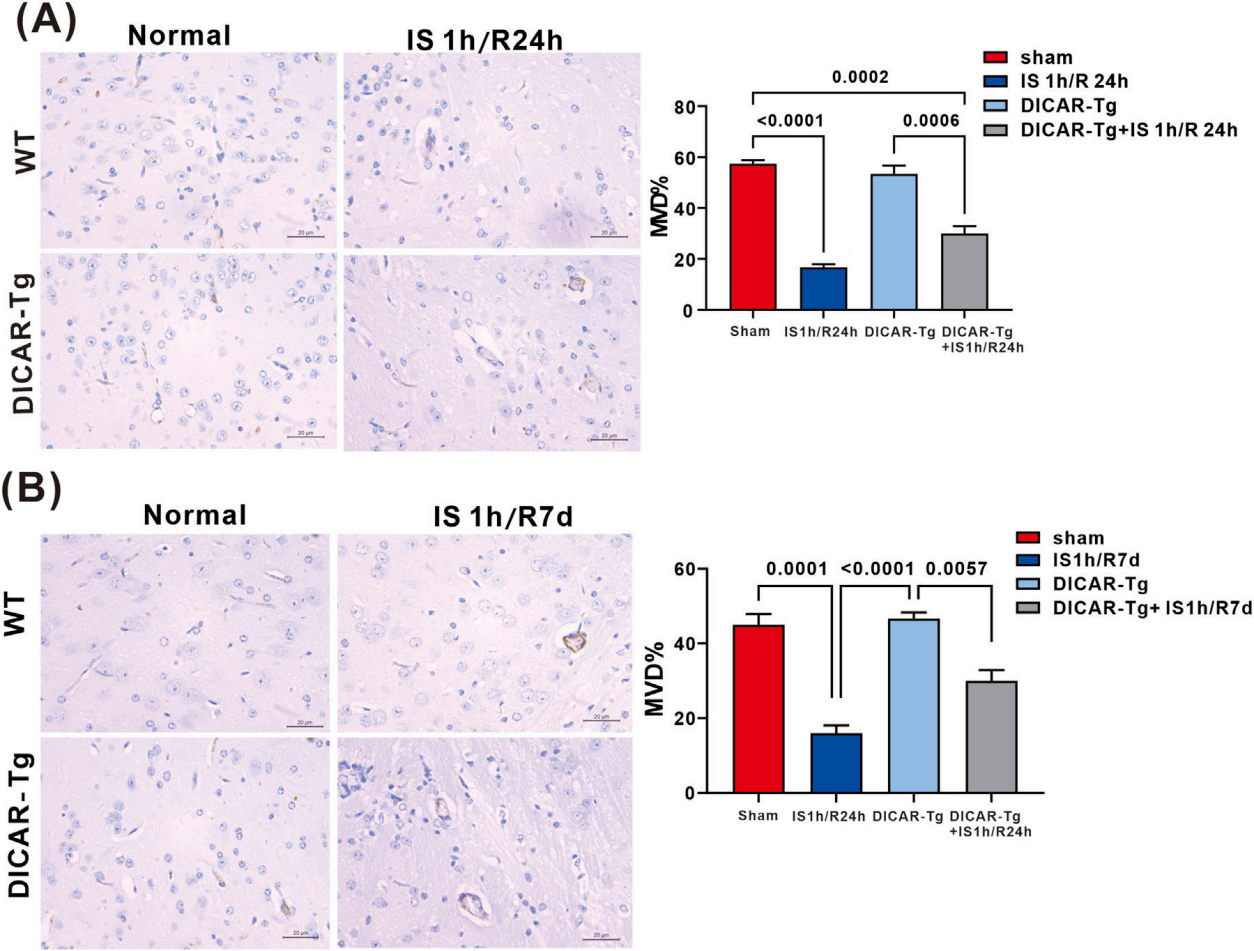
Anginogenesis ameliorated in brain IS/R model. **(A)** The representative images and microvascular deficiencies summary of VEGFR2 expression of brain IS1 h/24R detected by IHC; **(B)** the representative images and microvascular deficiencies summary of VEGFR2 expression of brain IS1 h/7d detected by IHC. Values represent the mean ± SD (n = 6 for each group).

Immunohistochemical analysis was performed to quantify VEGFR2 protein expression and microvascular density, a hallmark indicator of angiogenesis. Comparative histomorphometric analysis revealed a significant reduction in cerebrovascular density compared with the control groups (p < 0.0001), whereas DICAR-transgenic models exhibited notable neovascularization (p < 0.0001). Immunoblotting revealed differential regulation of VEGFR2 signaling pathways among the experimental groups. As depicted in Figure 2A, the levels of VEGFR2 protein were markedly reduced in WT mice subjected to ischemia–reperfusion injury, in contrast to the increase observed in DICAR-Tg mice (p < 0.0001). This persisted throughout the course of IS/R pathology, with similar expression patterns observed on days 1 and 7 post-ischemic intervals (Figure 2B).

### DICAR attenuates the miR-361-5P/PRMT signal pathway in hCMEC/D3 impairment by stroke

**FIGURE 3:**
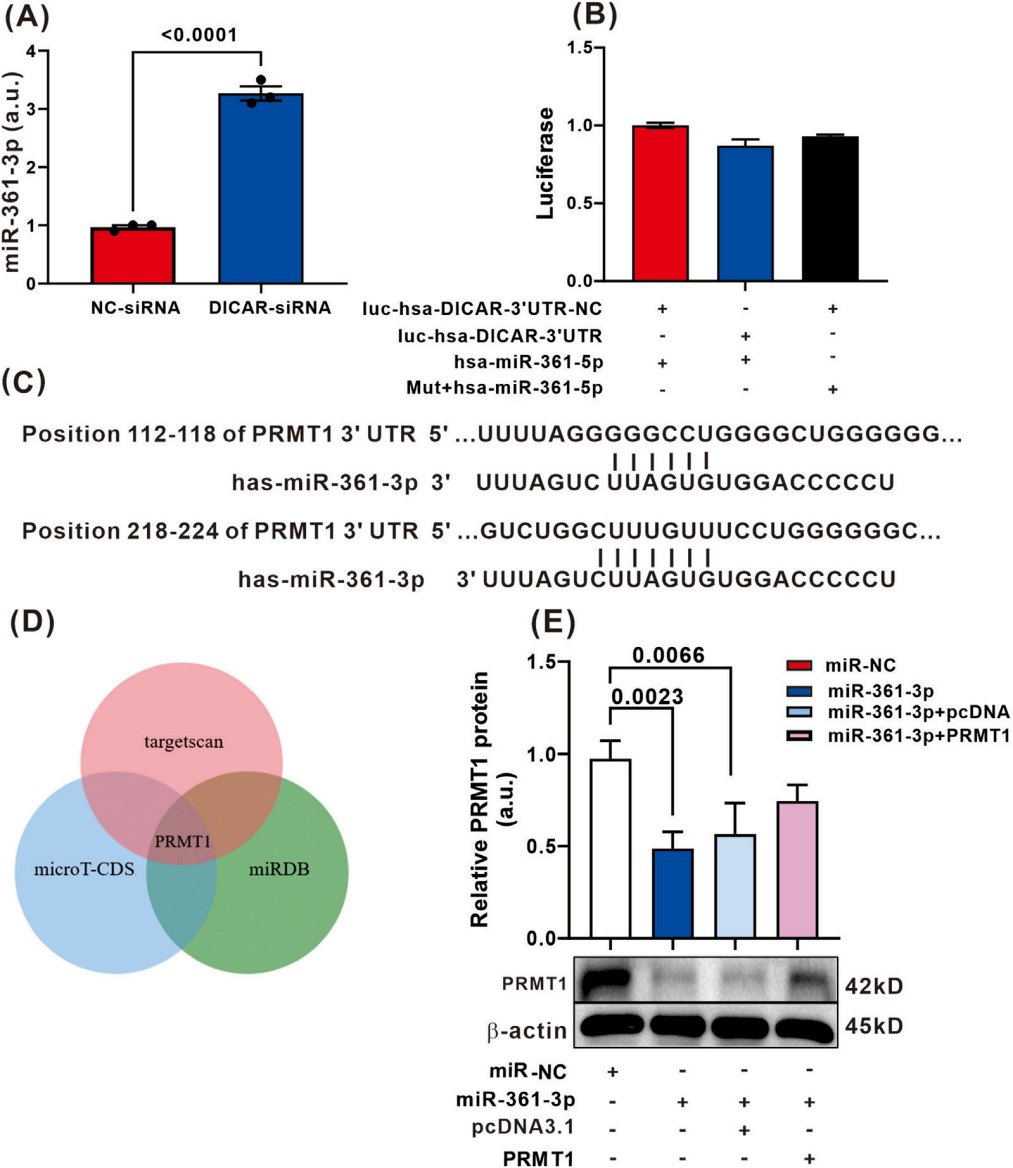
miR-361-5p/PRMT involved the function of DICAR on angenogenesis. **(A)** miR-361-5p expression regulated by DICAR-siRNA. **(B)** Luciferace detected the sponge function of DICAR on miR-361-5p. **(C)** Bioinformatics information analysis the binding sited of miR-361-5p and PRMT. **(D)** WB detected PRMT expression. Values represent the mean ± SD (n = 3 for each group).

A bioinformatics analysis identified DICAR as a putative molecular sponge for miR-361-5p, prompting our hypothesis that miR-361-5p mediates the angioregulatory function of DICAR. Transfection of DICAR-specific siRNA into hCMEC/D3 ECs resulted in DICAR knockdown (p < 0.001), concomitant with a significant increase in miR-361-3p expression (Figure 3A). To validate the direct molecular interactions, we engineered a dual-luciferase reporter system, which revealed no evidence of sequence-specific binding between DICAR and miR-361-3p (Figure 3B). This suggests that DICAR modulates miR-361-3p expression through indirect signaling mechanisms rather than canonical sponge activity.

Complementary bioinformatic analyses predicted the conserved binding capacity of miR-361-5p for protein arginine methyltransferase 1 (PRMT1) mRNA (Figure 3C), which was corroborated by consensus target identification across three independent databases (TargetScan, miRDB, and DIANA-microT; Figure 3D). PRMT1 is a predominant mammalian arginine methyltransferase that catalyzes monomethylation and asymmetric dimethylation of protein substrates. It contributes to vascular EC proliferation, differentiation, and angiogenic programming (Ishimaru et al., 2017; He et al., 2018).

Functional studies indicated that miR-361-5p overexpression significantly suppressed PRMT1 protein levels (p < 0.001), whereas PRMT1 reconstitution rescued miR-361-5p-mediated angiogenic inhibition (Figure 3F). Considered together, these results indicate that DICAR exerts pro-angiogenic effects on cerebral ischemia through the modulation of the miR-361-5p/PRMT1 regulatory axis, independent of direct miRNA sponging mechanisms. Results also indicate that DICAR interference may significantly inhibit the expression of miR-361-5p. This suggests that downregulating DICAR expression induced by glucose-oxygen deprivation indirectly regulates the expression of miR-361-5p in cells. Furthermore, our *in vitro* cell studies demonstrated that a miR-361-5p mimic suppresses PRMT protein expression. Other studies indicate that PRMTs mediate ECs function.

### DICAR-JP protects angiogenesis in hCMEC/D3 cells impaired by OGD/R *in vitro*

**FIGURE 4:**
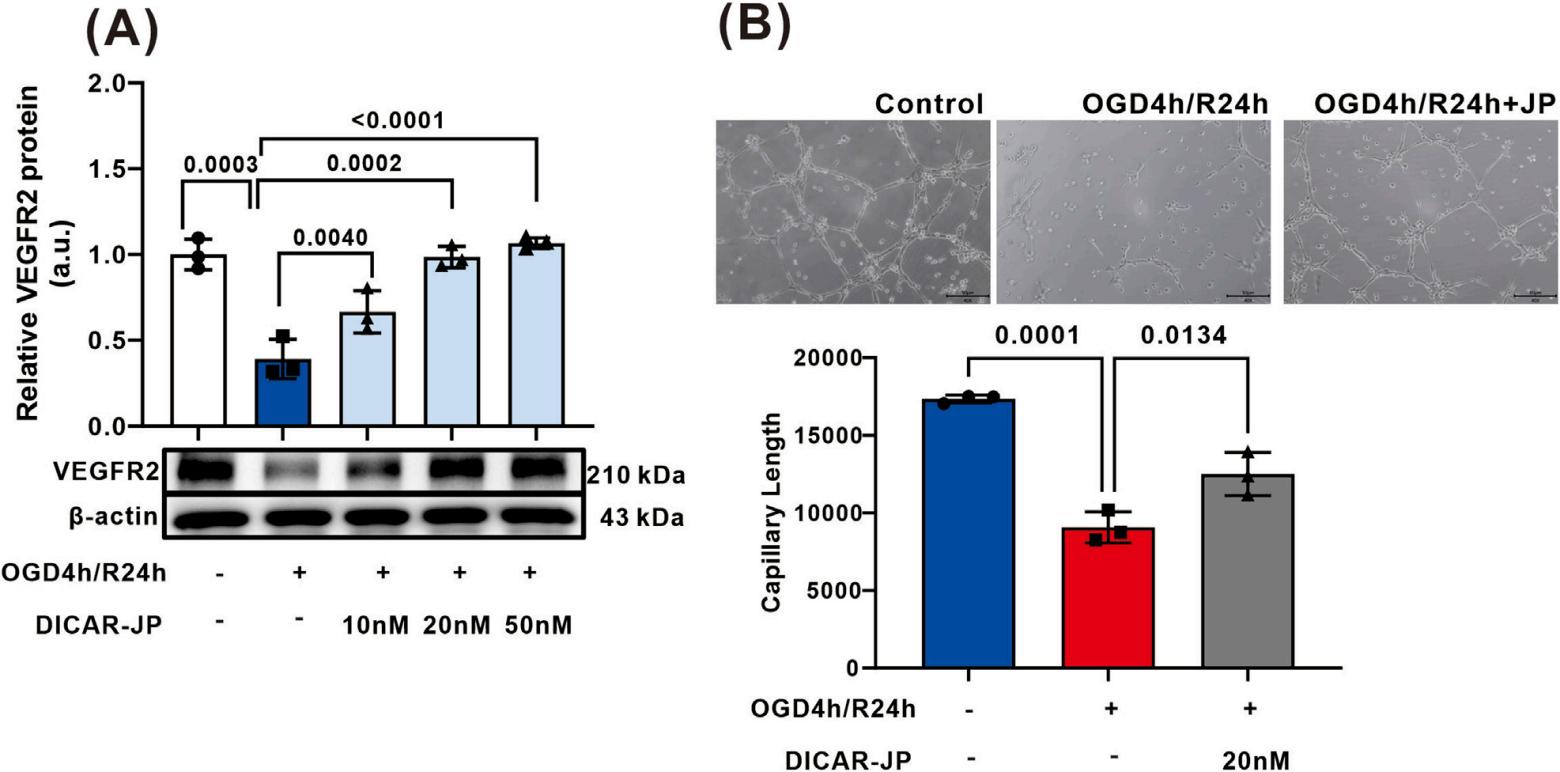
DICAR-JP reversed hCMEC/D3 angenogenesis formation impaired by OGD/R. **(A)** VEGFR2 protein expression detected by WB in hCMEC/D3; **(B)** the representative images and summary of tube formation. Values represent the mean ± SD (n = 6 for each group).

hCMEC/D3 cells were cultured to mimic brain vessels and transfected with DICAR-JP at various concentrations. As depicted in Figure 4A, VEGFR2 protein expression was significantly decreased in hCMEC/D3 cells, impaired by OGD/R. DICAR-JP reversed VEGFR2 protein expression in a dose-dependent manner. Additionally, we determined the effect of DICAR-JP on angiogenesis in hCMEC/3 cells using tube formation assays. Tube formation was impaired by OGD/R (p < 0.0001), and DICAR-JP (20 nM) treatment for 24 h significantly ameliorated this effect (p < 0.0134) (Figure 4B).

### AAV9-DICAR-JP protects the brain from ischemia in a mouse stroke model

**FIGURE 5:**
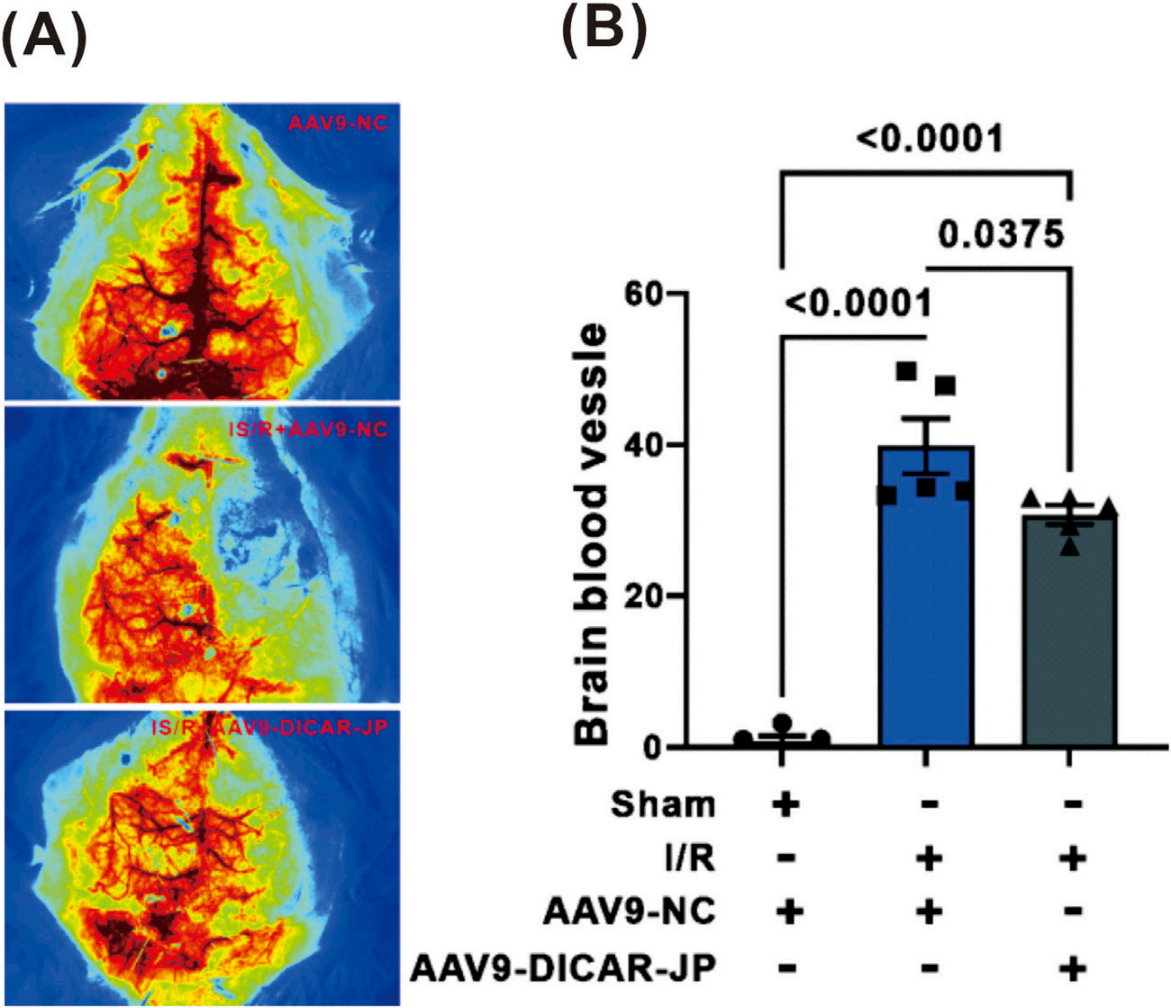
AAV9-DICAR-JP ameliorated brain blood flow of mouse broke. **(A)** Presentative images of blood flow. **(B)** The summary data of blood flow. Values represent the mean ± SD, n = 5 for each group.

Following the identification of DICAR-JP as the functional domain responsible for the activity of DICAR, we hypothesized that this structural motif mediates its cerebroprotective effects during ischemic stroke pathogenesis. Therefore, we established a standardized middle cerebral artery occlusion/reperfusion (MCAO/R) murine model, followed by the stereotaxic administration of AAV9-DICAR-JP constructs for sustained 14-day cerebral expression before ischemic induction. Laser speckle contrast imaging revealed that DICAR-JP transduction significantly ameliorated cerebral hypoperfusion, restoring 68.3% ± 5.2% of baseline cortical perfusion versus 42.1% ± 6.8% in the scrambled RNA controls (p < 0.001; Figures 5A,B). The therapeutic efficacy was further corroborated using dynamic contrast-enhanced MRI, which showed enhanced microvascular integrity in DICAR-JP-treated cohorts (Figures 5A,B). These results collectively establish DICAR-JP not only as a functional domain, but a novel therapeutic candidate that exerts nuclear-targeted cerebrovascular protection through ischemia-modulating mechanisms.

## Discussion

In the present study, we found that 1) DICAR overexpression inhibits brain impairment induced by stroke; 2) DICAR regulates the miR-361-3p/PRMT1 signaling pathway, thereby influencing angiogenesis in brain ECs; and 3) AAV9-DICAR-JP plays an important role in vascular remodeling following brain impairment.

Promoting angiogenesis is a focal area of research in stroke therapy (Wu et al., 2025). ECs and progenitor ECs are involved in angiogenesis during stroke (Kleeberg et al., 2025). Enhancing angiogenesis increases blood flow and the oxygen supply to ischemic areas, thereby protecting the damaged tissue. The newly formed vascular network exhibits strong permeability, substantially improving the blood supply to the perihematomal region and facilitating the removal of hematoma metabolites to promote absorption (Severson et al., 2025). Subsequently, new vessels appear around and penetrate the hematoma starting from the 7th day after collagenase-induced IS (Elmously et al., 2022; Li et al., 2025). A large number of clinical studies have shown that oxidative stress plays a key role in the pathological progression of ischemic stroke (Zhou et al., 2021, Li et al., 2025). This vicious cycle is characterized by an imbalance between excessive reactive oxygen species (ROS) production and endogenous antioxidant capacity (Su et al., 2020), which not only directly participates in the occurrence and development of neuronal injury during the acute phase (Li et al., 2025), but also synergistically acts with inflammatory responses to exacerbate blood-brain barrier disruption and ischemic brain damage (Li et al., 2025). Reactive oxygen species generated from mitochondrial dysfunction are a key factor triggering pyroptosis in ischemic stroke (Franke et al., 2021, Wang et al., 2025). In brain microvascular endothelial cells, mitochondrial ROS (mtROS) promotes NLRP3 inflammasome activation through the PPARα/GOT1 axis, thereby initiating a caspase-1-dependent pyroptosis cascade (Wang et al., 2025). Additionally, mtROS can also induce various pathological processes such as endoplasmic reticulum stress, ion flux disorders, and lysosomal rupture, all of which are closely related to the initiation of pyroptosis. In our previous research, we have confirmed that DICAR be a anti-pyroptosis circRNA, and this suggested that DICAR belongs induced angiogenesis induced anti-pyroptosis. In the present study, we found that DICAR overexpression promotes angiogenesis in a mouse model of cerebral ischemia. Hematologic compression of the vasculature results in ischemia and hypoxia of the local brain tissue, which in turn stimulates angiogenesis. DICAR is a powerful circular RNA that ameliorates brain stroke by promoting angiogenesis.

Moreover, miR-361-3p alleviates cerebral ischemia–reperfusion injury by targeting NACC1 through the PINK1/Parkin pathway (Ye et al., 2022). It was recently demonstrated that miR-361-3p regulates cementoblast differentiation through Erk1/2 and PI3K-Akt signaling (Jin et al., 2024; Liao et al., 2019). However, miR-361-3p also reportedly plays a role in metabolism associated with cardiovascular diseases (Qi et al., 2025; Ye et al., 2022). In pulmonary arterial hypertension, downregulation of miR-361-3p drives pathological vascular remodeling by promoting the aberrant proliferation of pulmonary arterial smooth muscle cells (Zhou et al., 2022). Mechanistically, miR-361-3p overexpression suppresses this process by inhibiting the cyclin D1/CDK4 axis, a key regulator of cell cycle progression in vascular pathologies (Zamarbide Losada et al., 2023; Wang et al., 2020). In diabetic foot ulcer, miR-361-3p expression is inversely correlated with CSF1R expression. Functional studies suggest its regulatory role in macrophage-mediated inflammatory responses through the CSF1R/PI3K/AKT signaling axis, which modulates wound healing dynamics associated with diabetic complications (Jin et al., 2024). We used a bioinformatics analysis to identify a relationship between miR-361-3p and DICAR. We then transfected DICAR-siRNA into cells. Although miR-361-3p was upregulated by DICAR-siRNA, results of luciferase reporter assays indicated that there was no direct relationship between DICAR and miR-361-3p. In addition, we found that miR-361-3p could bind to the 3′-UTR of PRMT1. Moreover, miR-361-3p overexpression downregulated PRMT1 protein expression. PRMTs regulate tumorigenesis, metastasis, and drug resistance through arginine methylation, which involves processes such as cell cycle regulation, DNA damage repair, and epithelial-mesenchymal transition (Ning et al., 2023). PRMT inhibition ameliorates symptoms associated with spinal muscular atrophy (SMA) by targeting neuroinflammation, suggesting its potential as a standalone or adjunctive treatment.20 The imbalance between PRMT1 and DDAH (dimethylarginine dimethylaminohydrolase) is associated with hyperglycemia-induced endothelial dysfunction. Pharmacological agents, such as telmisartan, can improve endothelial function by modulating the PRMT1/DDAH II pathway (Gray et al., 2010).

Based on our previous characterization of DICAR-JP as a functional nucleic acid sequence within DICAR (Yuan et al., 2023), we demonstrated its cardioprotective effect in attenuating diabetes-induced cardiomyocyte pyroptosis through valosin-containing protein (VCP)-mediated ubiquitination and subsequent degradation of Med12. Although the anti-pyroptotic mechanism of DICAR-JP has been established, its therapeutic potential in vascular pathophysiology remains unexplored until the present study. We also confirmed that AAV9-DICAR-JP, when injected for 14 days, plays a protective role in the brain.

In conclusion, we demonstrated that the DICAR/miR-361-5p/PRMT signaling pathway is involved in angiogenesis in brain ischemia/reperfusion (I/R), and DICAR-JP is a candidate target nucleic acid drug for promoting vascular remodeling during brain I/R.

## Data Availability

The raw data supporting the conclusions of this article will be made available by the authors, without undue reservation.
