# Correction: DICAR/DICAR-JP exerts therapeutic effects in brain stroke via the miR-361-5p/PRMT1 pathway

**DOI:** 10.3389/fphar.2025.1756716

**Published:** 2025-12-08

**Authors:** Zhijun Yu, Aihua Jiang, Kai Yang, Lin Zhan, Zifei Xiang, Li Chen, Zheng Kuai, Qiong Yuan

**Affiliations:** 1 Pharmacy, Medical College, Wuhan University of Science and Technology, Wuhan, China; 2 Hubei Province Key Laboratory of Occupational Hazard Identification and Control, Medical College, Wuhan University of Science and Technology, Wuhan, China; 3 Key Laboratory of Medical Electrophysiology, Ministry of Education & Medical Electrophysiological Key Laboratory of Sichuan Province Collaborative Innovation Center for Prevention of Cardiovascular Diseases, Institute of Cardiovascular Research, Southwest Medical University, Luzhou, China; 4 Department of Geriatrics, Zhongshan Hospital, Fudan University, Shanghai, China

**Keywords:** stroke, diabetes-induced circulation-associated circular RNA, RNA functional domain, angiogenesis, miR-361-5p, PRMT1

Affiliation “2 Hubei Province Key Laboratory of Occupational Hazard Identification and Control, Medical College, Wuhan University of Science and Technology, Wuhan, China” was erroneously assigned to authors Zhijun Yu, Aihua Jiang and Kai Yang as a Present Address. Instead the affiliation is now linked to only author Zhijun Yu who was affiliated with the institution at the time of the study.

Author Kai Yang was erroneously assigned to affiliation 1 Pharmacy, Medical College, Wuhan University of Science and Technology, Wuhan, China. The correct affiliation for author Kai Yang is affiliation 3 Key Laboratory of Medical Electrophysiology, Ministry of Education & Medical Electrophysiological Key Laboratory of Sichuan Province Collaborative Innovation Center for Prevention of Cardiovascular Diseases, Institute of Cardiovascular Research, Southwest Medical University, Luzhou, China.

Authors Aihua Jiang and Kai Yang were erroneously omitted as co-first authors.

The original article has been updated.

