# Supplementary material for: DICAR/DICAR-JP exerts therapeutic effects in brain stroke via the miR-361-5p/PRMT1 pathway
Source: Front Pharmacol. 2025 Nov 27;16:1721188. doi: 10.3389/fphar.2025.1721188 (PMC12722810; doi:10.3389/fphar.2025.1721188)
Supplement: Supplementary file 1 [file Table1.docx]

**Table S1 Translation sequences**

| **Gene name** | **Sequence** |
| --- | --- |
| DCIAR-JP | CAACCTCCGGGGCCACAATAGCGAGATTTGTAAGACTCCAGGGCCTCCCAG |
| miR-361-3p mimic | Sense: 5’-UCCCCCAGGUGUGAUUCUGAUUU-3’  Antisense: 5’-AUCAGAAUCACACCUGGGGGAUU-3’ |

**Table S2**

| **Gene name** | **Primer sequences** |
| --- | --- |
| Mouse GAPDH | Forward: 5'-CACTGAGCAAGAGAGGCCCTAT-3'  Reverse: 5'-GCAGCGAACTTTATTGATGGTATT-3' |
| Mouse DICAR | Forward: 5'-TACTATGAAGAGGGATGGTTGG-3'  Reverse: 5’-AAATGGCACTTGATATGTTTGTT-3' |
