# Supplementary figures and images for: DICAR/DICAR-JP exerts therapeutic effects in brain stroke via the miR-361-5p/PRMT1 pathway
Source: Front Pharmacol. 2025 Nov 27;16:1721188. doi: 10.3389/fphar.2025.1721188 (PMC12722810; doi:10.3389/fphar.2025.1721188)

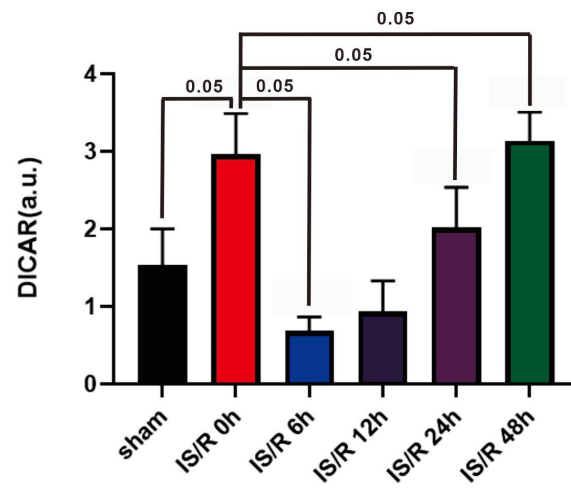

**Figure S1 DICAR expression in brain tissue of IS/R model. N = 3.**

Supplement: Supplementary file 2 [file Image1.pdf]
